# Evidence for a Potential Role of Metallothioneins in Inflammatory Bowel Diseases

**DOI:** 10.1155/2009/729172

**Published:** 2009-08-26

**Authors:** Anouk Waeytens, Martine De Vos, Debby Laukens

**Affiliations:** Department of Gastroenterology, Ghent University, De Pintelaan 185, 9000 Gent, Belgium

## Abstract

Inflammatory bowel diseases (IBDs) are a group of chronic, relapsing, immune-mediated disorders of the intestine, including Crohn's disease and ulcerative colitis. Recent studies underscore the importance of the damaged epithelial barrier and the dysregulated innate immune system in their pathogenesis. Metallothioneins (MTs) are a family of small proteins with a high and conserved cysteine content that are rapidly upregulated in response to an inflammatory stimulus. Herein, we review the current knowledge regarding the expression and potential role of MTs in IBD. MTs exert a central position in zinc homeostasis, modulate the activation of the transcription factor nuclear factor (NF)-*κ*B, and serve as antioxidants. In addition, MTs could be involved in IBD through their antiapoptotic effects or through specific immunomodulating extracellular effects. Reports on MT expression in IBD are contradictory but clearly demonstrate a deviant MT expression supporting the idea that these aberrations in IBD require further clarification.

## 1. Introduction

Metallothioneins (MTs) are a superfamily of small proteins that are present in virtually every living organism [1]. A typical feature is their highly conserved number and position of cysteine residues, enabling them to incorporate monovalent and divalent metal atoms and to reduce reactive oxygen and nitrogen species [2]. So far, 19 human isoforms have been cloned, 11 of which are known to be functional (i.e., MT1A, MT1B, MT1E, MT1F, MT1G, MT1H, MT1M, MT1X, MT2A, MT3, and MT4) [3]. The majority of these genes cluster together on a single locus on chromosome 16 (16q13) [4]. Heterogeneity of isoforms results from posttranslational modifications and/or variations in metal composition [5]. The most widely expressed isoforms are MT-1 and MT-2, and they are highly inducible [3]. On the basal level, MT-2 appears to be expressed more than the MT-1 isoforms. The MT-3 and MT-4 proteins are constitutively expressed and are found mainly in the brain [6], kidney [7], and reproductive organs [8] (MT-3) and in certain squamous epithelia [9] (MT-4). Human MT isoforms are regulated independently of each other and can be induced by metals, stress hormones, cytokines, reactive oxygen species (ROS), and chemicals [3]. In mice, the situation is more simple. Only four functional murine MT genes are known (MT1, MT2, MT3, and MT4), and the MT1 and MT2 isoforms are coordinately regulated [5, 10].

## 2. MT Regulation in Inflammation

One of the most striking observations that link MTs to inflammation is their rapid upregulation in response to a variety of stresses including inflammation. Hepatic expression of MTs is dramatically elevated in response to bacterial infection, an effect mediated by endotoxin (lipopolysaccharide-LPS). This observation has lead to the classification of MTs as acute phase proteins. LPS induction of MT gene expression in mice occurred in each organ examined (liver, kidney, pancreas, intestine, lung, heart, brain, ovary, uterus, and spleen) [11]. This induction by LPS was shown to be mediated by several proinflammatory cytokines, including interleukin (IL)-1, IL-6, tumor necrosis factor (TNF)-*α*, interferon (IFN)-*γ* [11], nitric oxide (NO) [12] as well as the stress hormones glucocorticoids [13]. These factors have been shown to be able to upregulate MTs independently of LPS. They seem to act synergistically and result in different levels of MT expression, depending on the tissue and the combination of factors [11, 14–16]. ROS generated during the inflammatory response may activate MT expression through multiple pathways, including directly by stimulating an antioxidant response element and specific metal response elements in the promoter region as well as indirectly by events associated with second-messenger protein kinase pathways [17, 18]. During radiation injury to the small intestine, which implicates ROS and leads to acute inflammation, metallothioneins were induced [19]. All these data illustrate that MT regulation in inflammation is a rapid but complex process with different results in different tissues. One constant, however, is that MT induction by inflammatory mediators seems to be conditional upon the presence of zinc [5].

## 3. MT Functions Relevant in Inflammation and Possibly in Inflammatory Bowel Diseases (Figure 1)

The first function of MTs that was put forward as playing a role in inflammation was their central position in zinc homeostasis, redistributing the intracellular zinc pool. In that way, MTs can have two opposite roles: they can either increase the intracellular zinc pool, thereby facilitating metabolic processes during the acute phase response or sequester zinc to allow maximal activity of enzymes which would be otherwise inhibited by zinc [5]. Clarifying these postulations is hampered by the immense number of enzymes that use or are inhibited by zinc. Furthermore, the affinity of MTs for zinc probably differs depending on the stimulus by which MTs are induced. In an in vivo displacement assay of cadmium (whose affinity for MT is stronger than that of zinc), it was shown that oxidative stress-induced MT displaced zinc to cadmium, on the other hand, MT induced by fasting stress or by restraint stress did not [20]. This study did not make a distinction between different MT isoforms, although metal-binding capacities of isoforms can vary [21, 22], as can their gene-regulation [23, 24]. Conformational changes of MT under certain stimuli might also explain the observed influence on zinc affinity. It has been shown that the binding of ATP (which reflects the energy state of a cell) to MT elicited conformational changes and altered zinc binding in MT [25]. 

Zinc deficiency is a potential complication of Crohn's disease and may result from a variety of processes, including reduced dietary intake, impaired absorption, increased excretion, hypoalbuminemia, or an internal redistribution of zinc [26, 27]. Although the consequences of this deficiency on the pathogenesis of the disease are not clear [28], it could be implicated through the involvement of zinc in immune function, redox signalling, and wound healing [29–32]. Zinc is crucial for the normal development and function of cells mediating innate immunity, that is, neutrophils, macrophages, and natural killer cells [33]. Phagocytosis, intracellular killing, chemotaxis, and oxidative burst are all negatively affected by zinc deficiency. In Crohn's disease, a defective innate immune response is more and more accepted as a pathogenic mechanism [34]. Zinc also has antiinflammatory properties. An important target herein is nuclear factor (NF)-*κ*B, a transcription factor that has a pivotal role in immune and inflammatory responses and as such also in IBD. Effects of zinc on NF-*κ*B activity have been attributed to its influence on the expression of the zinc-finger protein A20 that sequesters NF-*κ*B in the cytoplasm and inhibits IL-1- and TNF-*α*-induced activation of NF-*κ*B [35]. In vitro, zinc enhances the upregulation of A-20, thus decreasing NF-*κ*B activation and leading to decreased gene expression and generation of TNF-*α*, IL-1*β*, and IL-8 [36]. Taking these findings into account, a lack of intracellular zinc ion bioavailability may hamper the inhibition of NF-*κ*B, with subsequent maintenance of chronic inflammation.

The gene expression of IL-2, IL-12, and IFN-*γ* (T helper type 1 or Th1 cytokines) is zinc dependent, whereas T helper type 2 (Th2) cytokines in general are not affected by zinc deficiency [33]. As a consequence, an imbalance of Th1/Th2 cytokines appears in an experimental model of human zinc deficiency with a decrease of Th1 cytokines and a shift toward a Th2 phenotype [37]. This observation stands in contrast with the excessive Th1 cell response in the inflamed mucosa of Crohn's disease patients with active disease. However, in Crohn's disease patients with *inactive* disease, hyposecretion of IFN-*γ* is reported [38, 39]. Even though a relationship with zinc status has not been examined in these studies, zinc deficiency could be at the basis of this observation.

Studies where zinc was administered to rats or mice with chemically induced colitis showed a dose-dependent therapeutic effect [40–44]. Furthermore, zinc was shown to induce MT synthesis in ileal and colonic mucosa of control rats and to a lesser extent in that of colitic rats [45]. A placebo-controlled double-blind cross-over trial was conducted with seven Crohn's disease patients and seven ulcerative colitis patients that had inactive to moderately active disease and received oral zinc supplementation [46]. Although supplementation increased plasma zinc concentrations and slightly (but not significantly) increased mucosal MT concentration, there were no changes in histological inflammation or disease activity. However, it seems that zinc supplementation can resolve permeability alterations in patients with Crohn's disease in remission and as such reduce the risk of relapse in Crohn's disease [47]. This observation is supported by data that show a positive effect of exogenous zinc on intestinal repair in vitro [30] and on tight-junction permeability in experimental colitis [42]. Although the precise mechanism is not understood, it appears to be independent of MTs [48].

Next to their role in zinc homeostasis, MTs are reported to modulate the activation of NF-*κ*B. However, published results vary concerning the relationship between MT expression level and NF-*κ*B activity. The modulatory effect might be based upon a direct interaction [49], regulation of zinc concentrations [50], or modulation of the redox balance through antioxidant functions [51]. Data supporting a positive regulatory role for MTs on NF-*κ*B activity are the zinc-induced inhibition of this activity. Through sequestration of zinc, MTs can attenuate this zinc-induced inhibition and activate NF-*κ*B [50]. The requirement of MTs for the expression of macrophage colony stimulating factor, a chemokine downstream of NF-*κ*B, is another illustration of this positive regulatory role [52]. On the other hand, data exist that MTs may function as a negative regulator of NF-*κ*B, showing that MTs inhibited the activation of NF-*κ*B by TNF-*α* [53, 54] and that splenocytes from MTnull mice displayed elevated levels of NF-*κ*B activity [55]. Although these reports seem contradictory, explanations for this discrepancy could be found in the distinct redox regulation of NF-*κ*B activation between the cytoplasm and the nucleus [51], a balance which may be modulated by the antioxidant capacities of MTs. Another explanation might be found in differences in the cell types used. Although it seems obvious that a (negative or positive) correlation exists, MT effects can also be independent of NF-*κ*B. For example, a protective effect of MTs on acute inflammatory lung injury was not mediated via NF-*κ*B dependent pathways [56]. Thus, to determine the exact role of MTs in the NF-*κ*B pathway in IBD, NF-*κ*B activity should be investigated in vivo in experimental models.

As just mentioned, the antioxidant capacities of MTs might influence the inflammatory response through modulating NF-*κ*B activity, but a more obvious role for MTs as antioxidants is the sequestration of harmful oxygen and nitrogen intermediates which are generated during the inflammatory response. In order to kill bacteria and parasites, infiltrating neutrophils and macrophages produce free oxygen radicals (hydrogen peroxide, NO, and superoxide anion) which are extremely cytotoxic to host cells [57, 58]. MTs can, together with other known molecules such as superoxide dismutase, vitamin E, and ascorbate, provide a cytoprotection for host cells, preventing cellular damage and allowing survival and growth in an inflammatory environment [59]. The increased presence of ROS, an imbalance in antioxidant expression, and oxidative DNA and protein damage have been reported in IBD [60–63]. In Crohn's disease, oxidative damage as measured by lipid peroxidation correlated inversely with the concentration of MTs [61]. ROS can disrupt the epithelial barrier function by destabilizing tight junctions [64], thus increasing permeability, a phenomenon which is observed in patients with Crohn's disease [65]. Experiments in animal models of IBD have already confirmed the possibility to use antioxidants as therapeutic agents [66–69]. In IL-10 deficient mice, local mucosal administration of the antioxidant enzyme superoxide dismutase (SOD) by genetically modified *Lactobacillus* bacteria significantly reduced the severity of inflammation [70]. Mice overexpressing human SOD demonstrated attenuated inflammation when subjected to a mild form of dextran sodium sulphate- (DSS-) induced colitis and a remarkable survival benefit from severe DSS colitis [71].

In certain oxidative and inflammatory environments MTs have been shown to reduce apoptosis [72], and in cases where inflammation-dependent apoptosis is detrimental, induction of MTs might provide a benefit. Since Crohn's disease is characterized by defective T cell apoptosis, whereas T cells from ulcerative colitis patients show a strong activation induced apoptosis, the role of MTs might be different in these two diseases. MTs have also been accredited antimicrobial properties [73, 74], which might be another relevant feature given the defective interaction of the host with the mucosal flora in IBD. Macrophages isolated from MTnull mice showed a significantly lower bactericidal effect on *Staphylococcus aureus* than macrophages from control mice. Furthermore, LPS- and TNF-*α*-stimulated MT-null macrophages produced less NO than those from control mice, which was due to reduced activity of inducible NO synthase [73]. One MT isoform that was isolated and purified from housefly larvae had direct antibacterial effects on *Escherichia coli* as assayed by the plate growth inhibition assay [74].

Under certain conditions such as cell proliferation, differentiation, and after cell injury, MTs are translocated from the cytosol to the nucleus. MT regulation during cell cycle progression has been demonstrated in normally cycling cells, with maximal nuclear accumulation within the S and G2 phases. High cytoplasmic expression occurred during late G1 and G1/S transition, and basal amounts were found in the G0 phase [75, 76]. Hepatocytes show a transient nuclear localization of MTs at the G1-to-S phase transition during the priming phase of liver cell regeneration after partial hepatectomy [77]. Two premises for the nuclear retention of MTs have been proposed. First, it might reflect the role of MTs as chaperones to provide zinc for crucial enzymes and transcription factors involved in cell division [78]. Otherwise, it has been proposed that it might protect DNA from oxidative damage [79].

Beside their intracellular functions, MTs could also be involved in inflammation and IBD through specific extracellular effects [80]. For example, MTs have been shown to directly and specifically mediate leukocyte chemotaxis [81]. Extracellular MTs can stimulate lymphocyte proliferation [82]. MT binds to the plasma membrane of both T and B lymphocytes, but, in the absence of a costimulatory agent, MT induces lymphoproliferation only in B cells. MT also enhances the capacity of naive B lymphocytes to differentiate into plasma cells [83]. On the other hand, MTs can suppress cytotoxic T cell function in vitro and a T-dependent humoral response in vivo [84–86].

## 4. MT Expression in IBD

Given their possible functions in IBD pathogenesis, the expression of MTs has been studied in patient samples by different research groups. These studies have yielded contradictory results (Table 1) and as such the role of MTs in IBD is not yet clarified. First of all, two studies reported an increase in MT expression in IBD [87, 88], while the rest demonstrated a downregulation. In most studies no differences were reported between Crohn's disease and ulcerative colitis, except in two studies (both using DNA microarrays) where opposite findings were found, that is, decreased MT expression in ulcerative colitis and normal expression in Crohn's disease [89] versus normal expression in ulcerative colitis and increased MT expression in Crohn's disease [88]. The influence of medication is also not clear yet, given that one study reports an influence of steroid therapy on MT expression [90] whereas a second study contests this [91]. A study describing downregulation of MTs in vitro in colon epithelial cells after stimulation with azathioprine did not discuss this effect in patients, although two azathioprine-treated patients were included in the study [88]. The results of this study should be interpreted cautiously, considering the small dataset used. Finally, the immunohistochemical studies do not agree whether MT expression is confined to the epithelium [92–94] or whether expression in the lamina propria occurs as well [87, 90, 91]. The study of MT protein expression in IBD is even complicated by the possible destructive influence of the oxidative environment on the immunodominant epitope of the protein. Therefore, it is not sure whether the absence of immunoreactivity equals absence of MT protein or whether it reflects a failure of the antibody to recognize the present protein. 

The inconsistencies between the various studies could be explained by different sampling. MT expression could depend on the grade of local inflammation but also on the intestinal region, that is, ileum versus colon. Therefore, an ileal sample that has signs of mild inflammation and a piece of colonic tissue that suffers severe inflammation, both from a Crohn's disease patient, may possibly show different MT expression levels, although they will both be considered “Crohn's disease” samples. Therefore, careful sampling and precise classification of the samples could help in elucidating the exact MT expression pattern in IBD. Furthermore, patient characteristics such as age, medication, disease activity, zinc status, or even food intake before sampling could also influence MT levels and should therefore, if possible, be examined or at least mentioned in the study outline.

## 5. MTs in Experimental Colitis Models

The availability of MTnull mice (knockout for MT1 and MT2) and the use of recombinant MT have permitted the investigation of the involvement of MTs in animal models of inflammation. In the DSS model of colitis, MTs were not protective in two separate studies comparing MTnull mice with wild type mice [44, 94]. One of these studies even found that, after DSS administration, MTnull mice had a significantly *lower* disease activity index than had wild type mice [44], suggesting that MTs are rather unfavorable in DSS colitis. This finding was, however, not confirmed on histology. In disagreement with this postulation is the observation that, in the same study, administration of zinc as an MT-inducer resulted in a lower disease activity index and less histological damage. Nevertheless, it could not be proven that this effect was MT-dependent since no increase in colonic MT-levels was found following zinc-treatment. The second study did not provide disease activity indices and found no differences in histological colon damage between MTnull mice, wild type mice, and transgenic mice overexpressing MT1 in the intestine [94]. This study found a fourfold increase in total colon MT concentration after seven days of DSS administration, different from other studies in experimental colitis that found no changes [44] or a decrease in MT content [45] in DSS mice or in dinitrobenzene-sulphonic acid colitic rats, respectively. Apparently, the behaviour of MTs in experimental colitis models is as obscure as in human IBD.

## 6. Protective Functions of MTs in Other Animal Models of Inflammation

The role of MTs in models of other inflammatory conditions seems clearer and in most cases is a favorable one. In an animal model for multiple sclerosis, a chronic inflammatory and demyelinating disease of the central nervous system in which oxidative stress plays a pathogenic role, MTs were demonstrated to be protective [97]. MTnull mice were more susceptible to *Helicobacter pylori*-induced gastritis and showed more severe inflammation of the stomach than wild type mice [98]. This correlates with the reported antibacterial activity that was associated with MT function. This activity might be mediated directly by MTs themselves [74] or indirectly through nitric oxide production [73]. Endogenous MT protected against acute lung injury induced by LPS, especially against pulmonary oedema [56]. In the collagen-induced arthritis model, repeated administration of MT1 and 2 during the course of disease dramatically reduced the incidence and severity of the disease [99]. MTs suppressed the disease through the generation of IL-10- and transforming growth factor (TGF)-*β*-producing type 1 regulatory T-like cells [100]. In all of these models, MTs seem to be protective against local inflammation. In the TNF-induced lethal shock model (a model for systemic inflammatory response syndrome), however, MTs seem to sensitize [101]. An explanation for this finding was not found, although it is contradictory to the reported resistance that MTs confer to the cytotoxic effects of TNF in vitro [102] and to the protective effects of MTs in another model of systemic inflammatory response syndrome (LPS-induced lethal shock in sensitized mice) [103]. The antiinflammatory effects of MTs on the LPS-related organ damages could be mediated, at least in part, via the inhibition of the protein expression of proinflammatory cytokines (IL-1*β*, IL-6) and chemokines (granulocyte/macrophage colony-stimulating factor (GM-CSF), macrophage inflammatory protein (MIP)-1*α*, MIP-2, macrophage chemoattractant protein (MCP)-1, and keratinocyte chemoattractant (KC)) [104].

## 7. Conclusion

Although several functional associations of MTs can confer a role for this family of proteins in the pathogenesis of IBD, the results of human and experimental colitis studies are not decisive. However, it is clear that a deviant MT expression exists in this disease, and as such it is important to meticulously clarify these aberrations in Crohn's disease and in ulcerative colitis. Furthermore, investigating whether the regulation of MTs in these diseases is dependent or independent on inflammation will add knowledge on their involvement in IBD.

## Figures and Tables

**Figure 1:**
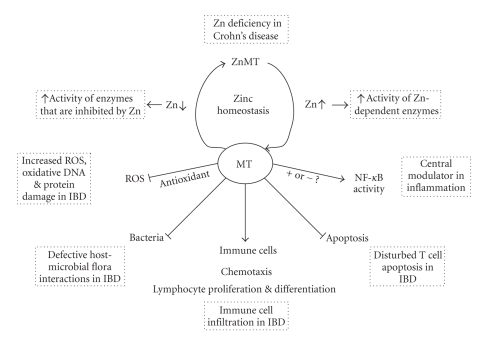
MT functions relevant in IBD. IBD is characterized by the presence of an increased level of ROS in the mucosal intestinal tissue as well as oxidative DNA and protein damage, defective host-microbe interactions, immune cell infiltration, and a disturbed T cell apoptosis. On all of these elements, MTs can have effects. In addition, MTs can have a dual role in enzyme activation through the release or sequestration of zinc. Finally, MTs are reported to regulate the activation of the transcription factor NF-*κ*B, which has a key role in inflammatory responses.

**Table 1:** MT expression in IBD patients. CD: Crohn's disease, UC: ulcerative colitis, IHC: immunohistochemistry, RIA: radio-immunoassay, Ag-hem: Silver-heme saturation assay, and qRT-PCR: quantitative reverse transcriptase-polymerase chain reaction.

Study	Subjects	Methods	Results
Clarkson et al. [90]	Ileal resection specimens of 13 CD patients (6 had received steroid therapy; 5 had not), 2 UC patients, and 3 controls	IHC on resection specimens	Less MT immunoreactivity in patients with IBD than controls; patients on steroid therapy had more immunoreactivity; immunoreactivity in enterocytes and lamina propria

Elmes et al. [91]	Ileal resection specimens of 17 CD patients (11 had received steroid therapy; 6 had not), and 5 controls	IHC on resection specimens	Decreased intestinal MT in IBD patients; no significant difference when patients had received steroid therapy; immunoreactivity in enterocytes and basement membrane region

Mulder et al. [95]	19 ileum and 16 colon specimens from 29 CD patients; 12 colon specimens and 1 ileum specimen from 12 UC patients; colon specimens from 18 control patients	RIA on homogenized mucosa (dissected from resection specimens)	MT content was decreased in noninflamed IBD mucosa compared with control mucosa; further decrease, was found in inflamed mucosa; no differences between UC and CD; no significant effect of medication or tissue localization

Sturniolo et al. [96]	Colonic biopsies of 24 UC patients and 10 controls	Ag-hem on biopsies	Reduced MT concentrations in patients with active disease as compared with controls and patients in remission; reduced MT concentrations in inflamed versus noninflamed mucosa taken from the same patient

Bruwer et al. [87]	22 CD patients, 48 UC patients, 10 controls	IHC on resection specimens	MT overexpression in the fibroblasts of all ulcerative and/or fissural lesions in UC and CD; MT overexpression in intestinal epithelial cells of 40% of UC and CD lesions correlated significantly with the grade of inflammation

Lawrance et al. [89]	Colonic resection specimens with moderately severe histological inflammation from 12 UC and 6 CD patients (with moderately severe clinical disease) and from 6 controls	DNA microarray	Decrease of MT1H and MT1G mRNA expression in UC; no difference in CD

Ioachim et al. [92]	Ileum, colon or rectum resection specimens from 10 CD patients, 41 UC patients, 5 controls	IHC	Decreased MT expression in UC and CD compared with normal mucosa; no difference in MT expression between UC and CD; in UC, a gradually decreased expression from remission, to resolving and to active phase was observed; only epithelial MT expression

Kruidenier et al. [93]	Resection specimens from 19 CD patients, 15 UC patients, 18 controls	RIA on tissue homogenates and IHC on resection specimens	RIA: Lower tissue MT content in inflamed CD and UC mucosa compared with noninflamed and control mucosa; IHC: decreased MT-positive epithelial cell numbers at inflamed sites in CD and UC patients; no detection of MT in lamina propria

Dooley et al. [88]	2 sets of colon samples: (a) control (1 uninvolved colon from CD patient), 1 CD patient, and 1 UC patient; (b) control, 1 azathioprine-treated CD patient, and 1 azathioprine-treated UC patient; drug-treated CaCo-2 cells	microarray and qRT-PCR with consensus primer sequences for multiple metallothionein genes.	Microarray: upregulation of MT1F, MT1G, MT1H in CD in tissue set (a); downregulation of MT1F, MT1H, MT1L in azathioprine-treated CaCo-2 cells; confirmed by qRT-PCR
